# Long-Term Follow-Up After Fecal Microbiota Transplantation via Freeze-Dried Capsules for Recurrent *Clostridioides difficile* Infection

**DOI:** 10.20411/pai.v11i1.868

**Published:** 2026-01-21

**Authors:** Michelle T. Hecker, Christian Rosero, Rafael Mendo-Lopez, Brigid M. Wilson, Maria M. Torres-Teran, Curtis J. Donskey

**Affiliations:** 1 Department of Medicine, Case Western Reserve University School of Medicine, Cleveland, Ohio; 2 Department of Infectious Diseases, The MetroHealth System, Cleveland, Ohio; 3 Infectious Diseases Division, University Hospitals Cleveland Medical Center, Cleveland, Ohio; 4 Geriatric Research, Education, and Clinical Center, VA Northeast Ohio Healthcare System, Cleveland, Ohio; 5 Research Service, VA Northeast Ohio Healthcare System, Cleveland, Ohio

**Keywords:** *Clostridioides difficile*, fecal microbiota transplantation, freeze-dried capsules, recurrence

## Abstract

**Background:**

Fecal microbiota transplantation (FMT) is a standard therapy for recurrent *Clostridioides difficile* infection (CDI). Limited information is available on the durability of response after FMT via freeze-dried oral capsules and on whether patients who fail an initial FMT can be successfully managed with repeated FMT.

**Methods:**

We conducted a retrospective cohort study of all patients undergoing initial FMT for recurrent CDI via freeze-dried capsules from March 2015 through June 2022 at 2 acute-care hospitals. Information on response to FMT during the initial management period (ie, 3 months after the initial FMT) and long-term durability of response was collected through direct communication with patients and medical record review. Episodes occurring within 90 days of the initial FMT were defined as recurrences, whereas those occurring more than 90 days after the initial FMT were defined as additional CDI episodes.

**Results:**

Of 129 patients with recurrent CDI treated with FMT via freeze-dried capsules, 114 (89%) had experienced 3 or more prior episodes of CDI. At 3 months after the initial FMT, 103 (80%) patients had no recurrence, 26 (20%) patients had 1 or more recurrences managed with 1 (n=21) or 2 (n=2) additional FMTs, and 3 (12%) were transitioned to CDI suppressive therapy. During subsequent long-term follow-up (median 182 weeks), 21 of the 126 patients (17%) who did not transition to suppressive therapy had additional episodes managed with CDI therapy only (n=9), CDI therapy and additional FMT (n=10), or suppressive CDI therapy (n=2).

**Conclusions:**

In a real-world setting with long-term follow-up, FMT via freeze-dried capsules was effective for the management of recurrent CDI. Repeated FMT procedures were effective for the management of patients with early failure after initial FMT and with additional episodes during long-term follow-up.

## INTRODUCTION

Fecal microbiota transplantation (FMT) is an effective therapy for recurrent *Clostridioides difficile* infection (CDI) [1]. The recent demonstration that FMT can be administered safely and effectively via oral capsules has been an important advance [2, 3]. In a systematic review and meta-analysis, FMT via oral capsules had an overall efficacy of 82.1% with similar results for frozen and lyophilized (freeze-dried) capsules [2]. A microbiome therapeutic composed of purified *Firmicutes* spores resulted in a significant reduction in recurrence of CDI at 8 weeks (12% vs 40% in the placebo group) [4] and 24 weeks (21% vs 47% in the placebo group) [5]. In 2023, the Food and Drug Administration approved this oral fecal microbiota product (VOWST) for prevention of CDI recurrence in adults.

One limitation of many studies of FMT is that the duration of follow-up has been relatively short, typically 8 to 24 weeks [1–4]. In recent reports, a durable response with no recurrence after several months of follow-up was achieved in 76% to 82% of FMT recipients, most of whom had received 1 FMT via colonoscopy [6, 7]. It is not clear if FMT via capsules provides a similar durability of response. Moreover, limited information is available on whether patients failing an initial FMT can be successfully managed with repeated FMT [8, 9]. Here, we examined the efficacy and durability of response to FMT via freeze-dried capsules in 2 tertiary care hospitals, including the frequency of repeated FMT procedures.

## METHODS

### Study Setting

We conducted a retrospective cohort study of all patients undergoing initial FMT for recurrent CDI via freeze-dried capsules from March 2015 through June 2022 at MetroHealth Medical Center and the VA Northeast Ohio Healthcare System. Patients with recurrent CDI were screened for eligibility to receive FMT by the Infectious Diseases physicians at each institution (MTH, CJD). Patients with chronic conditions associated with diarrhea (eg, inflammatory bowel disease, irritable bowel syndrome, chronic pancreatitis) were eligible for FMT if there was a clear increase in symptoms compared with their baseline and if symptoms improved with antibiotic treatment of CDI. Patients with only 2 episodes of CDI (ie, initial episode and first recurrence) were eligible for FMT if 1 or both episodes resulted in hospitalization and/or met criteria for severe CDI [10].

Both facilities used standalone nucleic acid amplification tests (NAATs) for diagnosis of CDI until 2018 and then switched to 2-step algorithms with an initial NAAT followed by use of an enzyme immunoassay for toxins A and B if NAAT-positive [10]. After 2018, FMT was offered regardless of whether the toxin assay, when performed, was positive or negative, if patients had a typical syndrome consistent with CDI (ie, diarrhea defined as 3 or more unformed bowel movements with no alternative explanation) that resolved with CDI therapy, a positive NAAT for *C. difficile*, and at least 1 recurrence. The rationale for providing FMT to toxin-negative patients was based on evidence that many patients with a positive NAAT but negative immunoassay for free toxin in our facilities have risk factors for and clinical presentations consistent with CDI [10]. Others have demonstrated that some patients with severe, complicated CDI may have NAAT-positive but enzyme immunoassay for toxin-negative test results [11, 12].

### FMT Protocol

The protocol for donor screening and FMT capsule preparation has been previously reported [3, 13]. A total of 5 donors were used during the study period, with 2 donors providing stool specimens used in greater than 75% of the FMT procedures (ie, donor 1 provided most specimens before December 2018, and donor 2 provided most specimens after December 2018). For all patients, acute CDI symptoms were either resolved or substantially improved with CDI treatment at the time of FMT. For vancomycin-treated patients, the dosage was reduced when feasible to a low maintenance dose while awaiting FMT (eg, 125 mg once daily). CDI therapy was discontinued 2 days before the FMT. Most patients at MetroHealth Medical Center received a pre-procedure magnesium citrate laxative, whereas none of those at the Cleveland VA Medical Center did. We administered ∼38 capsules (approximately 60 mg of freeze-dried stool enclosed in size 00 capsules) prepared from approximately 40 grams of stool. A previous evaluation demonstrated that each capsule contained ∼8.5 log_10_ colony-forming units (CFU) of anaerobes, predominantly *Clostridium* spp. and *Bacteroides* spp., with lower concentrations of aerobic and facultative organisms [3]. For most patients, the entire dose was taken under supervision in the outpatient clinic. Patients were advised to contact their FMT providers with any concerns about symptoms. In addition, they were encouraged to contact their FMT providers and/or have their physicians contact the FMT providers for consultation regarding antibiotic prescriptions after the transplant [13].

All patients were contacted within 2-4 weeks after FMT to determine their response to the treatment and to assess for any potential adverse effects. Medical records were reviewed for any patients admitted to the hospital to assess whether any illnesses were possibly related to the FMT procedure. After reports of transmission of extended-spectrum beta-lactamase-producing *Escherichia coli* by FMT in 2019 [14] the donor stool specimens were screened for extended-spectrum beta-lactamase- and carbapenemase-producing bacteria, vancomycin-resistant enterococci, and methicillin-resistant *Staphylococcus aureus*.

Patients were informed that if the initial transplant was unsuccessful, additional FMTs might be required. Patients with recurrence of symptoms consistent with CDI (ie, diarrhea defined as 3 or more unformed bowel movements with no alternative explanation) were eligible for repeated FMT; nearly all patients receiving additional FMTs had a positive NAAT, but this was not an absolute requirement if symptoms were consistent with prior CDI episodes and persistent. Prior to the additional FMT, patients were treated with oral vancomycin or fidaxomicin to achieve symptom control, and the dosage was reduced to a low maintenance dose while awaiting FMT and discontinued 2 days before FMT.

### Evaluation of Initial and Durable Response to FMT

We collected baseline demographic and clinical data, including medical conditions and testing and treatment of prior CDI episodes. Follow-up data, including testing and treatment of subsequent CDI episodes, antibiotic use within 3 months after the first FMT, colectomy, and death, were collected by direct communication with patients and medical record review. The duration of follow-up was the time from first FMT to the last documented encounter with any provider in the medical record, last direct communication with FMT provider, or death, whichever occurred last. We defined the initial management period as the time from the first FMT to 3 months after the first FMT.

For this study, CDI episodes after FMT were defined as occurrences in which CDI treatment was prescribed for diarrheal symptoms (ie, 3 or more unformed stools per day) with or without confirmatory *C. difficile* laboratory testing. Episodes occurring within 90 days of the initial FMT were defined as recurrences, whereas those occurring more than 90 days after the initial FMT were defined as additional CDI episodes. We defined recurrences as episodes occurring within 90 days of the initial FMT based on evidence from whole-genome sequencing analysis that recurrence due to relapse of the same strain of *C. difficile* often occurs after the 8-week cut-off typically used to define recurrence for surveillance purposes [15].

### Outcomes and Data Analysis

The primary outcome was the percentage of patients with a recurrence or additional CDI episode at any time after the first FMT. The secondary outcome was the percentage of patients with an additional CDI episode after the initial 3-month management period. Separate Kaplan-Meier survival curves with 95% confidence intervals were estimated for the time to first CDI episode after the initial FMT and for the time to first CDI episode occurring after the end of the initial management period. The survival curves were presented with the number of patients at risk with event times censored at time of death, colectomy for CDI, initiation of suppressive therapy, or last medical follow-up. Fisher's exact test was used to compare the proportions of patients with no recurrence during the initial 3-month management period for subgroups of patients. Analyses were performed in R Version 4.4.1 using the survival package.

### Patient Consent Statement

The Institutional Review Boards of MetroHealth Medical Center and the Cleveland VA Medical Center deemed the study to be exempt from review because it was a case series describing routine patient management. The study does not include factors necessitating patient consent.

## RESULTS

During the study period, 129 patients (113 at MetroHealth and 16 at the VA Northeast Ohio Healthcare System) received their first FMT via freeze-dried oral capsules. Table 1 shows the characteristics of the patients. A total of 114 (89%) patients had experienced 3 or more episodes of CDI at the time of their first FMT. All patients previously received 1 or more courses of oral vancomycin, and 80% were receiving vancomycin up to 2 days prior to their first FMT. Forty (31%) of the 129 patients died during the follow-up period, but only 1 patient died due to CDI (ie, 1 patient died of fulminant CDI 347 days after initial FMT).

**Table 1.** Characteristics of 129 Patients Receiving Fecal Microbiota Transplantation (FMT) via Freeze-Dried Oral Capsules

Characteristics	n (%)
Female gender	77 (60)
Age, median (range)	72 (18-94)
Race	
White	117 (91)
Black	10 (8)
Other	2 (2)
End-stage renal disease	11 (9)
Diabetes mellitus	37 (29)
Active cancer within 6 months	8 (7)
Inflammatory bowel disease	9 (7)
Cirrhosis	2 (2)
Immunosuppressive medications	11 (9)
Number of CDI episodes prior to FMT	
2	15 (12)
3	51 (40)
>3	63 (49)
Enzyme immunoassay for toxin assay result prior to FMT	
Not done	81 (63)
1 or more positive	33 (26)
All negative	15 (12)
CDI treatment immediately prior to FMT	
Vancomycin	103 (80)
Fidaxomicin	25 (19)
Vancomycin and fidaxomicin	1 (1)
CDI treatment ever prior to FMT	
Standard vancomycin	122 (95)
Vancomycin pulse/taper	94 (73)
Fidaxomicin	58 (45)
Bezlotoxumab	2 (2)
Number of FMTs ever	
1	97 (75)
2	26 (20)
≥3	6 (5)
Death, all-cause*	40 (31)
Within 6 months of FMT	3
Within 1 year of FMT	15
Antibiotics within 3 months after first FMT	15 (12)

1 patient died of fulminant CDI 347 days after initial FMT

**Figure 1.**
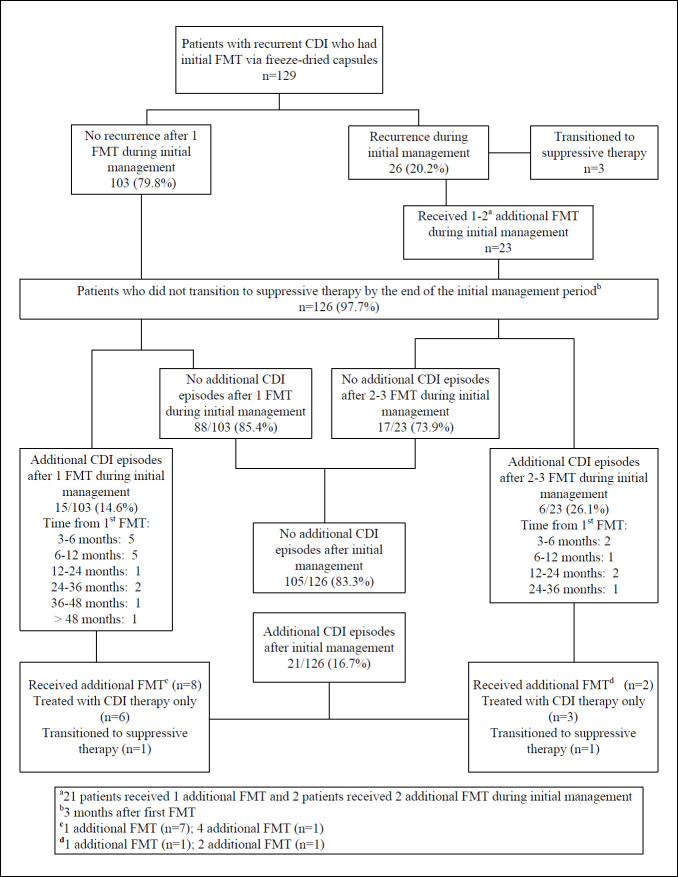
**Outcomes after fecal microbiota transplantation (FMT) via freeze-dried oral capsules for recurrent Clostridioides difficile infection (CDI) in 129 patients with recurrent CDI.** Patients receiving additional FMT during the initial management period and during subsequent long-term follow-up are shown in bold.

Figure 1 provides a flow diagram showing the outcomes of the patients. No patients reported adverse effects associated with the FMT procedure, and none developed bacteremia. After a single FMT, 103 (80%) patients had no recurrences at the end of the initial management period (3 months after the first FMT). Of the 26 (20%) patients with 1 or more recurrences during the initial management period, 23 (88%) received 1 (n=21) or 2 (n=2) additional FMTs, and 17 (74%) of those requiring additional FMT had no subsequent CDI episodes during long-term follow-up. Three of 26 (12%) patients with 1 or more recurrences during the initial management period were transitioned to CDI suppressive therapy with oral vancomycin 125 mg once daily. The 3 patients transitioning to suppressive therapy remained on lifelong oral vancomycin with no subsequent episodes of CDI.

Table 2 shows the percentages of patients with no recurrence at the end of the 3-month initial management period, stratified based on the number of prior CDI episodes, toxin testing results, and use of a pre-procedure laxative. Patients with only 2 prior episodes of CDI were less likely to have a recurrence by 3 months than patients with 3 or >3 prior episodes, but the differences were not statistically significant (*P ≥* .2). The percentages of patients with no recurrence by 3 months were similar for patients with negative versus positive toxin results (*P* = .4) and with versus without a pre-procedure laxative (*P* = .7).

**Table 2.** Percentages of *Clostridioides difficile* Infection (CDI) Patients With No Recurrence at the End of the Initial 3-Month Management Period Overall and for Subgroups of Patients

	No. with no recurrence/total no. (%)
Total	103/129 (79.8)
Subgroups	
Number of CDI episodes prior to FMT	
2*	14/15 (93.3)
3	41/51 (80.4)
>3	48/63 (76.2)
Enzyme immunoassay for toxin assay result prior to FMT	
Not done	61/81 (75.3)
1 or more positive	30/33 (90.9)
All negative	12/15 (80.0)
Use of a pre-procedure laxative	
Yes	58/74 (78.4)
No	45/55 (81.8)

FMT, fecal microbiota transplantation

* , patients with only 2 episodes of CDI (ie, initial episode and first recurrence) were eligible for FMT if 1 or both episodes resulted in hospitalization and/or met criteria for severe CDI

The median duration of follow-up after the first FMT was 182 weeks (range, 4-483). Of the 126 patients who did not transition to CDI suppressive therapy after the initial management period, 21 (17%) had 1 or more additional CDI episodes during the subsequent follow-up period. Additional episodes during the follow-up period occurred more frequently in patients requiring multiple vs a single FMT during the initial management period (6 of 23, 26% vs 15 of 103, 15%, respectively; *P* = .22). Overall, 47 of 126 (37%) patients had a recurrence or additional CDI episode at any time after the first FMT (ie, primary outcome), including 26 with recurrence during the initial 3-month management period and 21 with additional episodes after initial management.

**Figure 2.**
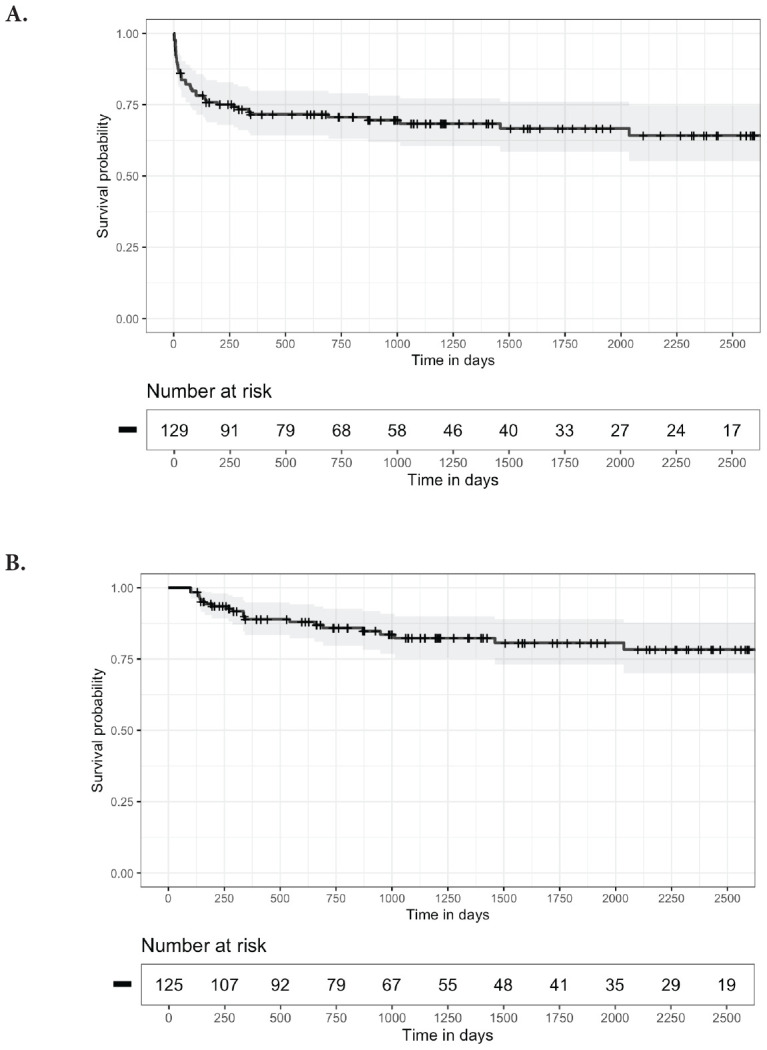
**Kaplan-Meier survival curves.** A) 95% confidence intervals estimated for the time to first *Clostridioides difficile* infection (CDI) recurrence after the initial fecal microbiota transplantation (FMT) and B) for the time to first CDI recurrence occurring after the end of the initial management period (3 months after the initial FMT), excluding patients with fewer than 3 months of follow-up (7 patients) or initiated on suppressive therapy during the initial management period (3 patients).

Figure 2 shows Kaplan-Meier Survival Curves for time to first recurrence of CDI after the initial FMT (A) and for the time to first CDI recurrence after the end of the initial management period (B). For 8 (38%) patients, the first additional CDI episode occurred more than 12 months after the first FMT. Twelve (57%) patients received systemic antibiotics within 3 months before the first additional episode. Additional CDI episodes were managed with CDI therapy only in 9 (43%), with CDI therapy and additional FMT in 10 (48%), and with suppressive CDI therapy in 2 (9%) patients.

## DISCUSSION

Our findings are consistent with previous evidence that FMT via freeze-dried capsules can be effective for initial management of recurrent CDI [2, 3]. No adverse effects were reported after the FMT procedures. For 80% of patients, there were no CDI recurrences within 90 days after a single FMT. This success rate is comparable to the 82% efficacy reported for oral capsules in a meta-analysis of 15 studies (5 using lyophilized material) at 8-26 weeks and the 84% efficacy reported for the microbiome therapeutic VOWST at 12 weeks [2, 5].

One novel aspect of our study is that we describe outcomes for patients who received multiple FMTs due to CDI recurrence within 3 months of a single FMT. Of the 26 patients with recurrence within 3 months after the first FMT, 23 (88%) received 1 or 2 additional FMTs, and of these patients, 17 (74%) had no additional CDI episodes during long-term follow-up. Paaske et al. [8, 9] also reported that a substantial proportion of patients failing initial FMT responded to repeat FMT procedures, resulting in overall cure rates of 79% to 81%. Thus, repeated FMT may be successful in a substantial proportion of patients with recurrence after a first FMT.

Our results also provide longer follow-up than previous studies of freeze-dried capsules. During a median follow-up of more than 3 years, 21 of 126 (17%) patients who had 1-3 FMTs during the initial management period had additional CDI episodes, and 10 (8%) received additional FMT procedures. Additional episodes occurred more frequently in patients requiring multiple vs a single FMT during initial management (26% vs 14%, respectively), and 38% occurred more than 12 months after the initial FMT. Fifty-seven percent occurred after receiving systemic antibiotics. Antibiotic exposure after FMT via colonoscopy has been identified as an independent predictor of loss of durability of FMT [6, 7].

Our study has limitations. The study was observational and did not include a concurrent control group. We relied on medical record review and patient interviews to obtain information, so some inaccuracy due to patient recall or inaccurate charting cannot be excluded. We cannot exclude the possibility that some patients might have cleared infection without receiving FMT. Previous studies have demonstrated that a substantial proportion of patients with multiple recurrences of CDI may be effectively treated with medical therapy alone [1, 2]. In 2 recent randomized trials, FMT was not more effective than placebo or tapering oral vancomycin therapy [16, 17], although some experts in FMT have suggested that methodologic issues in the recent placebo-controlled trial [16] might have contributed to the failure to demonstrate a benefit of FMT [18–21].

We did not assess the stool microbiome, so we cannot confirm that early engraftment of the FMT bacteria occurred. Such early engraftment has been demonstrated to be a predictor of CDI resolution after FMT [22]. We did not measure stool vancomycin levels to assess the potential for vancomycin to inhibit establishment of the transplanted bacteria. Inhibitory concentrations of vancomycin may persist in stool for 4-5 days after discontinuation of standard vancomycin regimens [23]. However, most patients received a pre-procedure magnesium citrate laxative, and our practice for vancomycin-treated patients was to manage with a relatively low maintenance dose while awaiting FMT. Finally, we did not require CDI testing to confirm recurrence in all cases with symptoms typical of prior CDI episodes prior to administering additional FMTs. Thus, we cannot exclude the possibility that some of these patients had alternative explanations for their diarrhea.

## CONCLUSION

In a real-world setting with long-term follow-up, FMT via freeze-dried capsules was effective for management of recurrent CDI. Repeated FMT procedures were effective for management of patients with early failure after initial FMT. During long-term follow-up, 17% of patients with successful initial management had additional episodes of CDI that resolved with additional CDI therapy with or without additional FMT procedures. Additional studies are needed to assess the efficacy of repeated FMT procedures in patients who fail initial FMT.
